# Morbidity burden and community-based palliative care are associated with rates of hospital use by people with schizophrenia in the last year of life: A population-based matched cohort study

**DOI:** 10.1371/journal.pone.0208220

**Published:** 2018-11-29

**Authors:** Katrina Spilsbury, Lorna Rosenwax, Kate Brameld, Brian Kelly, Glenn Arendts

**Affiliations:** 1 Centre for Population Health Research, Curtin University, Perth, Western Australia, Australia; 2 Institute for Health Research, The University of Notre Dame Australia, Perth Western Australia, Australia; 3 School of Occupational Therapy and Social Work, Curtin University, Perth, Western Australia, Australia; 4 Centre for Brain and Mental Health Research and School of Medicine and Public Health, University of Newcastle, Newcastle, New South Wales, Australia; 5 Centre for Clinical Research in Emergency Medicine, Harry Perkins Institute of Medical Research, University of Western Australia, Perth, Western Australia, Australia; 6 Department of Emergency Medicine, Fiona Stanley Hospital, Perth, Western Australia, Australia; University of Alberta, CANADA

## Abstract

**Objective:**

People with schizophrenia face an increased risk of premature death from chronic diseases and injury. This study describes the trajectory of acute care health service use in the last year of life for people with schizophrenia and how this varied with receipt of community-based specialist palliative care and morbidity burden.

**Method:**

A population-based retrospective matched cohort study of people who died from 01/01/2009 to 31/12/2013 with and without schizophrenia in Western Australia. Hospital inpatient, emergency department, death and community-based care data collections were linked at the person level. Rates of emergency department presentations and hospital admissions over the last year of life were estimated.

**Results:**

Of the 63508 decedents, 1196 (1.9%) had a lifetime history of schizophrenia. After adjusting for confounders and averaging over the last year of life there was no difference in the overall rate of ED presentation between decedents with schizophrenia and the matched cohort (HR 1.09; 95%CI 0.99–1.19). However, amongst the subset of decedents with cancer, choking or intentional self-harm recorded on their death certificate, those with schizophrenia presented to ED more often. Males with schizophrenia had the highest rates of emergency department use in the last year of life. Rates of hospital admission for decedents with schizophrenia were on average half (HR 0.53, 95%CI 0.44–0.65) that of the matched cohort although this varied by cause of death. Of all decedents with cancer, 27.5% of people with schizophrenia accessed community-based specialist palliative care compared to 40.4% of the matched cohort (p<0.001). Rates of hospital admissions for decedents with schizophrenia increased 50% (95% CI: 10%-110%) when enrolled in specialist palliative care.

**Conclusion:**

In the last year of life, people with schizophrenia were less likely to be admitted to hospital and access community-based speciality palliative care, but more likely to attend emergency departments if male. Community-based specialist palliative care was associated with increased rates of hospital admissions.

## Introduction

The average life expectancy of people living with schizophrenia in developed countries is around 20 years less than the general population [1, 2] although this varies by age of schizophrenia onset and gender. [3] This has been mostly attributed to premature death from cardiovascular disease, respiratory diseases, cancers and injury [4]. Factors associated with these excess early deaths include adverse side-effects of some antipsychotic medications [2]; under diagnosis of metabolic syndrome [5]; reduced rates of cancer screening [6] resulting in more advanced stages of cancer at diagnosis; high-risk lifestyle behaviours such as cigarette smoking [4, 7], alcohol and drug use [4]; and high levels of other medical comorbidity. [8] A review of cancer care in people with schizophrenia reported they experienced longer delays from diagnosis to treatment, were less likely to undergo surgery but had greater 30 day mortality post-surgery and received fewer chemotherapy and radiotherapy sessions. [9]

The episodic positive symptoms of schizophrenia such as delusions, thought disorder and hallucinations and the negative symptoms of apathy, social withdrawal, reduced self-care and cognitive dysfunction may act as barriers to accessing appropriate health care. [10, 11] A Canadian study reported that in the last six months of life, people with schizophrenia had more visits to general practitioners and psychiatrists but fewer visits to medical specialists, 27% less hospitalisations and were less likely to receive palliative care compared to a matched cohort. [12] A study from the United States reports that having any pre-existing psychiatric illness was associated with less acute care hospitalisations and intensive care but higher rates of ED presentations in the last 30 days of life and a greater likelihood of dying in a nursing home. [13] In contrast, a study of end-of-life care for mostly male US war veterans dying with cancer found those with schizophrenia received comparable care with similar proportions of patients who underwent surgical treatment for cancer, enrolled in hospice care and had advance directives and resuscitation orders in place, although a lower proportion of veterans with schizophrenia initiated chemotherapy. [14]

Community-based specialist palliative care delivered in the home or place of residence in the last year of life is associated with reduced presentations to ED [15, 16], reduced admissions to hospital [17, 18], shorter lengths of hospital stays [19, 20] and reduced hospital costs. [21] While most studies have focused on people dying with cancer, the evidence supporting the benefit of specialist palliative care in the setting of life-limiting non-cancer conditions is growing. [22, 23]

The aim of this study was to describe the trajectory of acute care health service use in the last year of life for people with schizophrenia and how this varied with morbidity burden, cause of death and access to community-based specialist palliative and non-palliative care. We hypothesised that people with schizophrenia would show low rates of community-based specialist palliative care in the last year of life, but that barriers to accessing acute care health services would be reduced in those who did.

## Methods

This was a population-based retrospective matched cohort study of acute health care service and community-based health care service use over the last year of life in people who died from 1 January 2009 to 31 December 2013 with or without a history of schizophrenia and aged 20 years and older in Western Australia (WA). Decedents with a lifetime history of schizophrenia, schizophrenia-like psychosis or schizoaffective disorders were eligible for inclusion and are referred to collectively as the schizophrenia cohort. Decedents in the schizophrenia cohort were identified from coded administrative data by searching all death registrations in WA from 2009 to 2013 and person-linked hospital admissions (1979 to 2013), emergency department (ED) presentations (2005 to 2013) and mental health registrations (1970 to 2013) using International Classification of Diseases (ICD) Version 9 [24] code 295 and ICD-10-AM codes F20, F21, F231, F232 and F25. [25] Data linkage and de-identified data extraction from the WA Data Linkage System was performed by the Data Linkage Branch at the WA Department of Health. In the absence of a unique personal identifier in Australia, the WA Data Linkage System uses probabilistic matching based on name and other identifiers. [26] Ethical approval to conduct this retrospective study on anonymized data with a waiver of the requirement to obtain consent was provided by the Human Research Ethics Committees at the WA Department of Health and Curtin University.

### Matching for population-based comparison cohort

Coarsened exact matching (CEM) was used to identify a matched comparison cohort of decedents without a history of schizophrenia. Matching was performed to create a similar balance in age groups, sex, partner status, indigenous status, residential location, relative social disadvantage and country of birth in both the schizophrenia and comparison cohorts. [27] Observations were weighted according to the size of the matching strata with unmatched population controls excluded. Matching and statistical analyses were adjusted for indigenous status, but we do report separately for this population subgroup.

### Causes of death

The Australian Bureau of Statistics uses ICD-10 to conduct multiple cause coding of all death certificates in Australia. The underlying cause of death is defined as “the disease or injury which initiated the train of morbid events leading directly to death…”, while other morbid conditions are classified as intermediate/intervening causes of death or contributory causes. [28] For the purposes of analysis we created cause of death groups from the reported leading causes of death overall in Australia [29]; most common causes of death in the cohort of decedents with schizophrenia; and less common causes of death that were over represented in the schizophrenia cohort compared to the matched cohort (S1 Table).

### Social and demographic variables

Marital status was classified as partnered (married or de-facto) or not/unknown at time of hospital admission, ED presentation and at time of death. Each decedent’s address was geocoded and used to assign accessibility categories based on the Australian ARIA+ index that takes into account road distance measurements to the nearest Service Centres and population size. [30] Socioeconomic status was estimated using quintiles of the Index of Relative Disadvantage (IRSD) which estimates the average disadvantage of small geographic areas. [31] Manual geocoding was also used to identify the type of residence at ED presentation, hospital admission and at death for decedents with schizophrenia and were classified as independent living (e.g. private residence); residential aged care facility (RACF); other care facility; no fixed address (e.g. homeless); and unknown/not stated.

#### Morbidity burden

Morbidity was defined as the presence of one or more of the 31 Elixhauser [32] conditions recorded in the primary or other 21 diagnostic fields during in-patient hospital stays over the last five years of life. [33] Morbidity burden was recorded as a temporal variable over the last year of life. For example, a decedent who had first mention of cancer on hospital admission at six months before death would have been recorded as having zero morbidity burden for the first six months of the last year of life before increasing to one after the cancer-related admission. We included psychoses in the calculation of total morbidity burden because this allowed us to differentiate health service use in decedents with schizophrenia before and after first mention of hospital admission for psychosis.

### Outcome measure: Emergency department presentations

Emergency department (ED) presentations were assigned daily per person and only the first ED visit per day was included in the time-to-event analysis. The ED presentations as part of inpatient hospital transfers were excluded. Available ED data included the triage category, hospital admission status, specific diagnosis and presenting symptom. Coded presenting symptom data and ICD-10 coded diagnosis data were only available for metropolitan based hospitals (approximately 70% of ED presentations). Comparative analyses involving coded ED presenting symptom and diagnosis data excluded non-coded ED presentations from the estimation process.

### Outcome measure: Hospital admissions

Hospital data were provided at the patient level in episodes of care. Multiple episodes of care were brought together into a single hospital admission when they included a statistical discharge (e.g. change of hospital care type but still in hospital) or transfer to another hospital. The principal diagnosis for the hospital admission was taken from the earliest episode of care when multiple episodes of care were present.

### Community-based care

The Western Australian Home and Community Care (HACC) Program is a Commonwealth and State Government funded program that provides support services to frail older people and younger people with disabilities and their carers for a nominal fee. Services provided included basic nursing care (e.g. wound dressings), personal care (e.g. showering), domestic assistance (e.g. shopping, house cleaning), assistance with maintaining social support, client care coordination, allied health (e.g. podiatry), group activity centres, transport and meals. The types of services accessed by decedents in the last year of life was available, but patterns of service use over the last year of life could not be described due to lack of reliable date information.

Silver Chain WA is the main provider of community-based specialist palliative care in WA, although the most comprehensive service is restricted to major metropolitan areas. A team of palliative care clinicians and nurses, allied health professionals and volunteers provide home nursing care, counselling, respite options, practical support and links to other services with the aim of enabling people with life-limiting illness to remain at home. A palliative nurse consultancy service is available to residential care facilities where client care is managed by registered nurses. A palliative care rural telephone advisory service provides specialist advice to local rural service providers 24 hours per day. Study data included enrolment and discharge dates into each Silver Chain service and the number and length of time of home visits. A client may have had multiple periods of enrolment if their condition was of a relapsing and remitting nature. Life-limiting conditions considered amenable to palliative care for this study were cancers, heart failure, renal failure, liver failure, chronic obstructive pulmonary disorder, motor neurone disease, Parkinson’s disease and Alzheimer’s disease as described previously. [34]

### Statistical analysis

Strata-weighted chi-square tests were used to assess equality of proportions. Differences between means were assessed with design-adjusted Wald tests. Multiple failure time-to-event analyses were performed to investigate the association of schizophrenia with acute care health service use in the last year of life. The multiple failure outcome events of interest were ED presentations and hospital admission any time in the one-year follow-up period. As cause of death is strongly associated with health service use, separate time-to-event analyses of the association of schizophrenia with health service use were performed for each cause of death. Decedents were excluded from the risk pool during periods of hospital stays.

A weighted kernel-density estimate of the hazard was used to graph unadjusted rates of ED presentations and hospital admissions over the last year of life. Time-to-event analyses were performed using flexible parametric Royston-Parmar models. [35] Variables that demonstrated temporal changes were treated as time-varying covariates including state of hospitalisation, number of comorbid conditions, partner status, residence in areas of relative disadvantage and level of accessibility to services. Standard errors were adjusted using the clustered sandwich estimator to account for correlated time between failure events. Matching strata weights were applied in all estimation procedures involving the matched comparison cohort. Variables used in the matching process were also included in regression analyses for full adjustment. The Sidak correction for multiple testing for each cause of death, assuming an alpha of 0.05 and 30 simultaneous comparisons, estimated a p-value of <0.0017 would demonstrate evidence of an association. Stata Statistical Software: Release 14 (Stata Corp, College Station, TX) was used.

## Results

Of the 63508 deaths in WA, 1196 (1.9%) of decedents had a lifetime history of schizophrenia. Decedents with schizophrenia were younger, lived in urban areas of more socioeconomic disadvantage, were more likely born in Australia and to have no partner at time of death compared to the general population of decedents (Table 1). A matched comparison cohort of 39265 decedents without a history of schizophrenia matched into 511 strata by age at death, sex, partner status at earliest record, indigenous status, residence in a major metropolitan area or not, socioeconomic disadvantage and country of birth was identified, each with a weighting relative to strata size. Two female decedents with schizophrenia could not be matched and were excluded from comparative analysis.

**Table 1:** Socio-demographic characteristics at time of death for decedents with schizophrenia compared to all other decedents who died over the same time five-year time period 2009–2013.

	All other decedents (n = 62312)		Decedents with schizophrenia (n = 1196)		Chi-squared test p-value
	N	%	N	%	
**Age at death (years)**					
20–29	972	1.6	51	4.3	
30–39	1352	2.2	88	7.4	
40–49	2508	4.0	130	10.9	
50–59	4728	7.6	170	14.2	<0.001
60–69	7790	12.5	212	17.7	
70–79	12550	20.1	228	19.1	
80–89	20924	33.6	241	20.2	
90+	11488	18.4	76	6.4	
**Sex**					
Male	32799	52.6	622	52.0	0.666
Female	29513	47.4	574	48.0	
**Index of relative social disadvantage**					
Most disadvantaged	16437	26.4	375	31.4	<0.001
More disadvantaged	13209	21.2	275	23.0	
Average disadvantage	10807	17.3	218	18.2	
Less disadvantaged	9578	15.4	148	12.4	
Least disadvantaged	8342	13.4	111	9.3	
Unknown	3939	6.3	69	5.8	
**Partner status**					
Not partnered	30498	48.9	981	82.0	<0.001
Partnered	31814	51.1	215	18.0	
**Accessibility index**					
Major cities	45430	72.9	919	76.8	<0.001
Inner regional	5297	8.5	58	4.8	
Outer regional	5154	8.3	91	7.6	
Remote	1723	2.8	44	3.7	
Very remote	820	1.3	16	1.3	
Unknown	3888	6.2	68	5.7	
**Born in Australia**					
No	26205	42.1	412	34.4	<0.001
Yes	36107	57.9	784	65.6	

Amongst decedents with schizophrenia, the demographic profile varied by sex. The average age at first schizophrenia record was 28.2 (standard deviation (SD) 8.1) years for males and 30.1 (SD 8.4) years for females (p = 0.099). A greater proportion of female decedents with schizophrenia (n = 265, 46.7%) were living in a residential aged care facility at time of death compared to males (n = 136, 22.4%), whereas a greater proportion of males were homeless or without a fixed address at time of death (n = 10, 1.6%) compared to females (<5, 0.2%). More male decedents with schizophrenia (n = 433, 70%) were born in Australia compared to females (n = 350, 61%). A greater proportion of female decedents with schizophrenia lived in urban areas (n = 457; 80% versus n = 447; 72%) while a greater proportion of males with schizophrenia died aged less than 30 years (n = 43; 7% versus n = 7; 1%).

### Causes of death

The most frequent principal causes of death in the schizophrenia cohort were cancers, ischaemic heart disease, intentional self-harm, chronic lower respiratory disease, dementias and accidental poisoning (Table 2). One third of decedents with schizophrenia had cancer recorded on their death certificate compared to more than half of all decedents in the matched cohorts. The exception was for breast cancer, where no difference between the cohorts was noted. The proportion of deaths in the schizophrenia cohort from diseases of the circulatory system were the same as that for the matched cohort. Amongst decedents with schizophrenia, a greater proportion of males had an underlying cause of death from intentional self-harm (n = 86, 14%) or accidental poisoning (n = 49, 8%) compared to females (n = 23, 4% and n = 15, 3% respectively, p<0.001).

**Table 2:** The leading causes of death for decedents with schizophrenia (n = 1194) compared against decedents in the matched control cohort (n = 39,265) by underlying cause or any mention of the cause on the death certificate.

Leading causes of death	Underlying cause of death^1^					Any mention on death certificate^2^				
	SCZ cohort		Matched cohort†			SCZ cohort		Matched cohort†		
	N	%	N	%	p	N	%	N	%	p
**Infectious diseases**										
Bacterial sepsis	17	1.4	347	0.9	0.018	69	5.8	2305	5.9	0.772
**Neoplasms**										
All cancers	199	16.7	11885	30.3	<0.001	218	18.3	13474	34.3	<0.001
Lung cancer	46	3.9	2423	6.2	0.001	47	3.9	2586	6.6	<0.001
Breast cancer	23	1.9	708	1.8	0.284	28	2.3	990	2.5	0.222
Of ill-defined sites	18	1.5	622	1.6	0.732	54	4.5	3028	7.7	<0.001
Colorectal cancer	17	1.4	1032	2.6	0.010	19	1.6	1222	3.1	0.005
Blood & lymph cancers	16	1.3	1076	2.7	0.010	20	1.7	1382	3.5	0.007
**Endocrine system**										
Diabetes	39	3.3	1206	3.1	0.465	145	12.1	4221	10.8	0.003
**Mental/behavioural**										
Dementias	65	5.4	2976	7.6	0.046	154	12.9	6160	15.7	<0.001
Schizophrenia	22	1.8	0	0.0	<0.001	208	17.4	0	0.0	<0.001
Due to use of alcohol	6	0.5	59	0.2	0.127	50	4.2	604	1.5	0.421
Due to use of drugs	6	0.5	30	0.1	0.276	39	3.3	192	0.5	0.002
Bipolar affective disorders	5	0.4	<5	0.0	<0.001	43	3.6	57	0.1	<0.001
Depressive episodes	<5	0.1	16	0.0	0.323	52	4.4	786	2.0	<0.001
**Nervous system**										
Parkinson's disease	13	1.1	307	0.8	0.005	26	2.2	693	1.8	<0.001
Epilepsy	9	0.8	65	0.2	0.035	23	1.9	281	0.7	0.043
**Circulatory system**										
Ischaemic heart disease	174	14.6	5836	14.9	0.205	277	23.2	10402	26.5	0.406
Cerebrovascular disease	53	4.4	2645	6.7	0.702	110	9.2	5298	13.5	0.665
Heart failure	25	2.1	760	1.9	0.013	138	11.6	5656	14.4	0.041
Hypertensive disease	12	1.0	470	1.2	0.412	119	10.0	5613	14.3	0.748
**Respiratory system**										
CLRD	70	5.9	1561	4.0	<0.001	137	11.5	3706	9.4	<0.001
Influenza, pneumonia	24	2.0	632	1.6	0.093	174	14.6	4855	12.4	<0.001
Pneumonitis due to solids/liquids	13	1.1	232	0.6	<0.001	61	5.1	1539	3.9	<0.001
Asthma	9	0.8	100	0.3	0.052	17	1.4	380	1.0	0.239
**Digestive system**										
Cirrhosis/liver disease	15	1.3	446	1.1	0.038	33	2.8	1240	3.2	<0.001
Intestinal obstruction	9	0.8	154	0.4	0.001	16	1.3	535	1.4	0.768
**Genitourinary system**										
Renal diseases/dialysis	16	1.3	701	1.8	0.970	90	7.5	4820	12.3	0.026
**Signs and symptoms**										
Ill-defined/ unknown	21	1.8	205	0.5	0.010	33	2.8	306	0.8	<0.001
**Injury and poisoning**										
Intentional self-harm	109	9.1	734	1.9	<0.001	109	9.1	716	1.8	<0.001
Accidental poisoning^3^	64	5.4	353	0.9	<0.001	96	8.0	661	1.7	<0.001
Accidental falls	16	1.3	655	1.7	0.866	22	1.8	892	2.3	0.695
Choking on food/liquid^4^	8	0.7	12	0.0	<0.001	9	0.8	24	0.1	<0.001
All other causes	154	12.9	6303	16.1						

^1^The disease or injury that initiated the train of morbid events leading to death.

^2^The number and proportion of decedents with this cause recorded anywhere on the death certificate (either underlying, antecedent or contributory cause of death).

^3^Includes poisoning by non-opioid analgesics, antipyretics, antirheumatics, narcotics, psychodysleptics, antiepileptic, sedative-hypnotic, antiparkinsonism or psychotropic drugs.

^4^ Inhalation and ingestion of food causing obstruction of respiratory tract.

†Strata weights applied and p-values estimated from design adjusted chi-square tests. SCH, schizophrenia; CLRD, chronic lower respiratory disease

The average total morbidity burden at the beginning of the last year of life was similar in the schizophrenia cohort and matched controls but the type of conditions varied (Table 3). Decedents with a lifetime history of schizophrenia had a higher proportion of uncomplicated diabetes, chronic pulmonary disease, neurological disorders, depression, hypothyroidism, obesity, alcohol and drug abuse and psychoses recorded in the five years prior to the last year of life relative to the matched cohort. The matched cohort had a significantly higher proportion of decedents with a history of cancer, circulatory disorders and liver disease at the start of the last year of life.

**Table 3:** The number of decedents with specific morbid conditions at one year before death in the schizophrenia and matched cohorts estimated from 5-year look back of hospital records.

Morbidity burden at one year before death	Schizophrenia cohort		Matched cohort†		
	N	Col %	N	Col %	p
**Elixhauser comorbid conditions**					
Psychoses*	294	24.6	77	0.2	<0.001
Hypertension, Uncomplicated	140	11.7	5410	13.8	0.045
Congestive Heart Failure	115	9.6	3689	9.4	0.786
Diabetes, Uncomplicated*	109	9.1	2521	6.4	<0.001
Chronic Pulmonary Disease*	105	8.8	2681	6.8	0.009
Alcohol Abuse*	97	8.1	2493	6.4	0.019
Depression*	82	6.9	1299	3.3	<0.001
Other Neurological Disorders*	80	6.7	1619	4.1	<0.001
Fluid and Electrolyte Disorders	75	6.3	2621	6.7	0.599
Drug Abuse*	68	5.7	823	2.1	<0.001
Cardiac Arrhythmias	67	5.6	3220	8.2	0.001
Renal Failure	64	5.4	2796	7.1	0.021
Solid Tumour Without Metastasis	59	4.9	5245	13.4	<0.001
Metastatic Cancer	56	4.7	4346	11.1	<0.001
Diabetes, Complicated	46	3.9	1695	4.3	0.446
Weight Loss	34	2.8	861	2.2	0.136
Liver Disease	33	2.8	1785	4.5	0.004
Obesity*	32	2.7	655	1.7	0.011
Peripheral Vascular Disorders	25	2.1	1459	3.7	0.004
Hypothyroidism*	24	2.0	359	0.9	<0.001
Valvular Disease	16	1.3	831	2.1	0.067
Deficiency Anaemia	16	1.3	771	2.0	0.129
Paralysis	15	1.3	487	1.2	0.964
Coagulopathy	15	1.3	804	2.0	0.060
Pulmonary Circulation Disorders	14	1.2	1003	2.6	0.003
Hypertension, Complicated	11	0.9	276	0.7	0.387
Rheumatoid Arthritis/Collagen Vascular	6	0.5	366	0.9	0.129
Lymphoma	<5	0.3	479	1.2	0.006
Blood Loss Anaemia	<5	0.3	182	0.5	0.525
Peptic Ulcer Disease Excluding Bleeding	<5	0.2	221	0.6	0.072
AIDS/HIV	<5	0.1	62	0.2	0.533
**At beginning of last year**					
Decedents with no comorbidity (n, %)	450	37.7	16054	40.9	0.119
No. conditions per decedent (mean, se)	1.4	0.05	1.3	0.01	0.013
**At time of death**					
Decedents with no comorbidity (n, %)	169	14.2	7084	18.0	0.002
No. conditions per decedent (mean, se)	3.7	0.1	3.5	0.02	0.036

No., number of conditions; Col, column *Overrepresented in decedents with schizophrenia

†Strata weights applied and p-values estimated from design adjusted chi-square tests. Values are those estimated as if the comparison cohort had the same age, sex, socioeconomic, partner, indigenous and country of birth structure as the schizophrenia cohort.

### ED presentations over the last year of life

ED presentations were more frequent for male decedents with schizophrenia compared to matched male controls (Table 4). Decedents with a history of schizophrenia tended towards less urgent ED presentations which were more likely to occur for social or behavioural symptoms or related to alcohol use relative to the matched comparison cohorts, with the difference more marked in males. A greater proportion of decedents with schizophrenia were transported to ED by police or correctional services compared to the matched cohort.

**Table 4:** Summary characteristics of ED presentations and hospital stays in the last year of life for decedents in the schizophrenia and matched comparison cohort stratified by sex.

	Female decedents					Male decedents				
In the last year of life	Schizophrenia n = 572		Matched† n = 20591		p	Schizophrenia n = 622		Matched† n = 18672		p
**ED presentations**										
Total cohort ED visits (n, row %)	1338	3.2	40708	96.8		2020	4.1	47293	95.9	
Decedents presented (n, col%)										
No	154	26.9	4493	23.9	0.114	170	27.3	5630	27.5	0.929
Yes	418	73.1	14305	76.1		452	72.7	14838	72.5	
										
ED visits/decedent (mean, se)	2.3	0.1	2.2	0.1	0.249	3.3	0.4	2.3	0.5	0.016
Transport to ED by police (n, col%)	38	2.8	128	0.3	<0.001	75	3.7	837	1.8	<0.001
Triage category (n, col%)										
Resuscitation	72	5.4	1982	4.8	0.183	89	4.4	3417	7.2	<0.001
Emergency	243	18.2	8077	19.8		348	17.2	9081	19.2	
Urgent	543	40.6	17374	42.7		712	35.2	18870	39.9	
Semi-urgent	411	30.7	11409	28.0		682	33.8	12325	26.1	
Non-urgent	67	5.0	1805	4.4		183	9.1	3513	7.4	
Presenting symptoms‡(n, col%)										
Pain	210	20.0	7509	24.5	<0.001	296	20.3	7295	22.1	<0.001
Respiratory	185	17.6	4944	16.1		223	15.3	4744	14.4	
Neurological	175	16.7	4932	16.1		195	13.4	4556	13.8	
Injury	88	8.4	2481	8.1		103	7.1	2776	8.4	
Social /behavioural	92	8.8	765	2.5		171	11.8	1230	3.7	
Drug/alcohol related	13	1.2	327	1.1		181	12.4	1236	3.8	
**Hospital admissions**										
Total cohort admissions (n,row %)	1828	1.7	108326	98.3		2162	1.9	113462	98.1	
Decedents admitted (n, col%)										
No	157	27.4	3556	18.9	<0.001	175	28.1	6094	29.8	0.410
Yes	415	72.6	15242	81.1		447	71.9	14373	70.2	
										
Admissions/decedent (mean, se)	3.2	0.4	5.8	0.2	<0.001	3.5	0.5	5.5	0.2	<0.001
Length of stay (mean, se)	7.7	0.4	5.1	0.1	<0.001	8.1	0.5	4.5	0.1	<0.001
Hospital stay care type (n, col%)										
Acute care	1727	94.5	104472	96.4	<0.001	2093	96.8	110299	97.2	<0.001
Rehabilitation	6	0.3	280	0.3		3	0.1	214	0.2	
Palliation	32	1.8	3122	2.9		41	1.9	2625	2.3	
Psychogeriatric care	41	2.2	237	0.2		16	0.7	173	0.2	
Maintenance	22	1.2	188	0.2		8	0.4	137	0.1	
Admissions (n, col %) involving										
Psychiatric care€	132	7.2	551	0.5	<0.001	204	9.4	725	0.6	<0.001
Emergency admission*¥*	995	54.4	34410	31.8	<0.001	1221	56.5	34751	30.6	<0.001
Stay in ICU	34	1.9	1600	1.5	0.193	64	3.0	2273	2.0	0.002
Ventilator support	25	1.4	905	0.8	0.018	42	1.9	1651	1.5	0.074

ED, emergency department; se, standard error; col, column; n, number; ICU, intensive care unit.

† Coarsened exact matching strata weights applied and p-values corrected to account for study design. Values are those estimated as if the comparison cohort had the same age, sex, socioeconomic, partner, indigenous and country of birth structure as the schizophrenia cohort.

‡ Estimated from the 70% of ED presentations coded with symptom and diagnostic information.

¥ Includes admissions via the ED and direct admissions to speciality care areas such as intensive care and burns units or via the ED of another hospital.

€ Hospital stay involved one or more days within a designated psychiatric unit.

**Fig 1:**
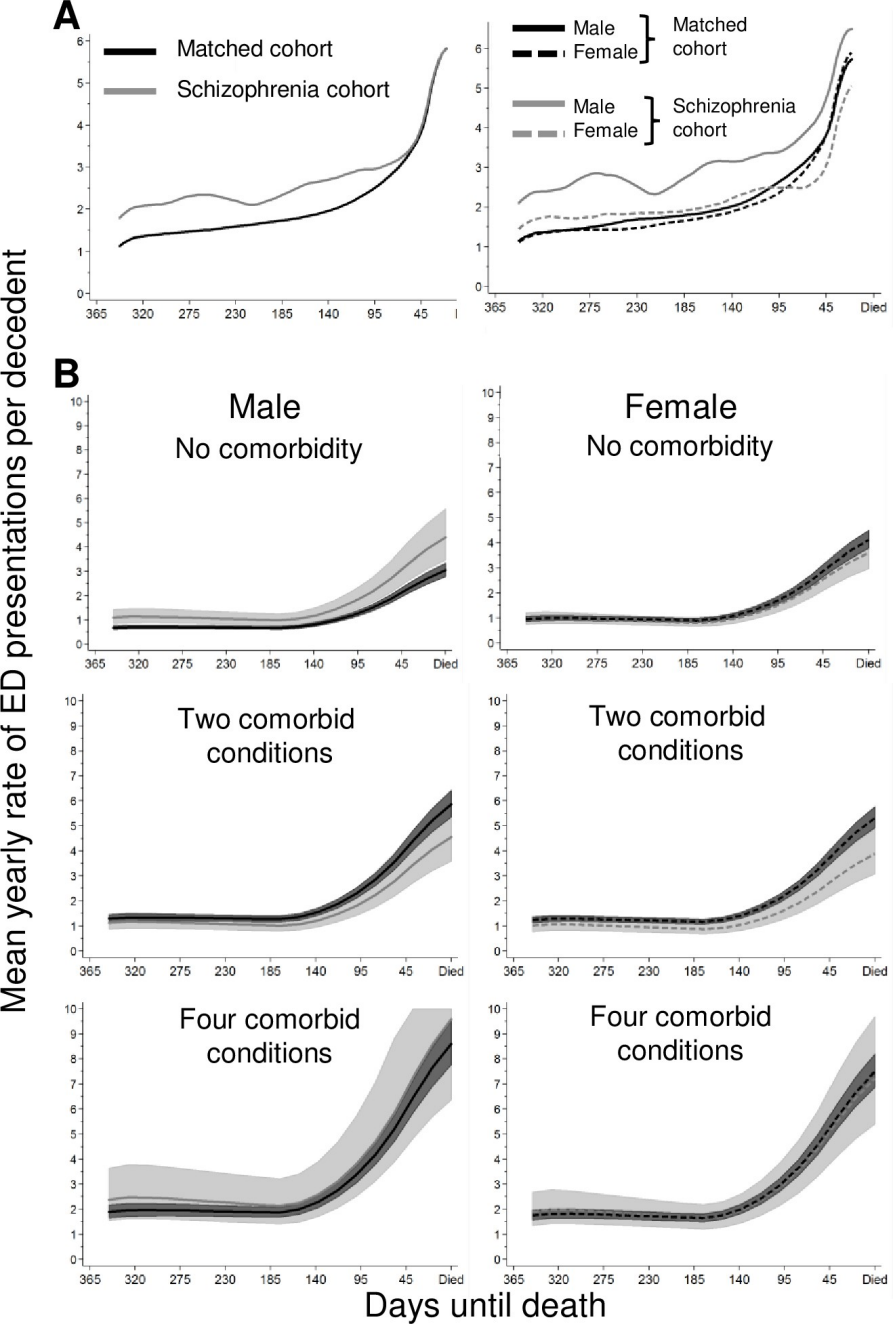
**The average yearly (hazard) rate of ED presentations per decedent over the last year of life for the schizophrenia and matched cohorts and stratified by sex, estimated from A) an unadjusted kernel-weighted estimator of the hazard and B) an adjusted flexible parametric proportional hazards model.** Values in B) predicted for Australian born non-Indigenous decedents living in a metropolitan area of average disadvantage without a partner, and aged 50 years or older at death. Sex and comorbidity were entered as interaction terms.

The rate of ED presentations was not constant over the last five years of life (Fig 1A). The unadjusted rate of ED visits over the last year of life was consistently higher in the schizophrenia cohort compared to matched cohort until the last two months of life when the rate of ED presentation of the matched cohort increased rapidly. When stratified by sex, most of the increased rate of ED presentations in schizophrenia appear to be driven by males.

After adjusting for confounders and averaging over the last year of life there was no difference in the rates of ED presentation between decedents with schizophrenia and the matched cohort (HR 1.09; 95%CI 0.99–1.19). However, testing for interaction terms indicated that the degree of association was strongly modified by gender and by the number of comorbid conditions (Fig 1B). Amongst male decedents with no comorbid condition, the rate of ED use was greatest in males with schizophrenia, whereas amongst female decedents with no comorbidity, the rate of ED use was highest amongst females in the matched cohort. With two comorbid conditions, the rate of ED presentations was lower for both female and male decedents with schizophrenia compared to their gender matched cohorts and by four comorbid conditions, there was no significant differences between the sexes or cohort in rate of ED presentations.

We investigated whether the rates of ED presentation between decedents in the schizophrenia and matched cohorts varied by causes of death (Table 5). Of all decedents who had cancer, choking on food/solids or intentional self-harm mentioned anywhere on their death certificate, decedents with schizophrenia tended towards higher rates of ED use in the last year of life compared to decedents from the matched cohort. Amongst decedents who died from intentional self-harm, 40.1% (n = 107) of ED presentations for people with schizophrenia were because of social/behavioural or drug/alcohol problems, compared to 26.9% (n = 806) of ED presentations for people without schizophrenia (p<0.001). Of all decedents who had ischaemic heart disease or respiratory disease recorded as a morbid event on their death certificate, there was no difference in the rates of ED use in the last year of life between those with schizophrenia and those in the matched cohort, except perhaps a trend of lower ED use in decedents with schizophrenia who died with influenza or pneumonia.

**Table 5:** Adjusted relative rates of ED presentations and hospital admission by decedents in the schizophrenia cohort compared to decedents in the matched cohort from multiple regression models for each cause of death listed anywhere on the death certificate.

Cause of death decedent subsets^¥^	ED presentations†			Hospital admission†		
	HR	95%CI	p	HR	95%CI	p
Bacterial sepsis	1.3	0.8–2.0	0.255	0.3	0.2–0.4	<0.001
**Neoplasms**						
All cancers combined	1.2	1.1–1.4	0.005	0.6	0.5–0.8	<0.001
Lung cancer	1.5	1.2–1.9	0.002	0.6	0.5–0.8	<0.001
Breast cancer	1.3	0.9–2.0	0.132	0.9	0.7–1.3	0.680
Colorectal cancer	0.8	0.4–1.7	0.590	0.5	0.3–1.0	0.037
Blood & lymph	0.8	0.5–1.3	0.432	0.3	0.2–0.5	<0.001
Ill-defined sites	1.4	1.1–1.8	0.018	0.6	0.5–0.8	0.001
**Endocrine system**						
Diabetes	1.0	0.8–1.2	0.767	0.5	0.3–0.9	0.014
**Mental/behavioural**						
Dementias	0.8	0.7–1.0	0.089	0.5	0.3–0.7	<0.001
Due to use of alcohol	0.8	0.5–1.1	0.160	0.3	0.2–0.5	<0.001
Due to use of drugs	1.3	0.8–2.2	0.264	0.5	0.3–0.9	0.017
Schizophrenia		NE			NE	
Bipolar affective disorders	0.9	0.6–1.3	0.479	0.5	0.2–1.1	0.082
Depressive episodes	0.7	0.5–1.1	0.093	0.5	0.3–0.8	0.007
**Nervous system**						
Epilepsy	1.0	0.5–1.7	0.874	0.7	0.4–1.3	0.273
Parkinson's disease	0.8	0.5–1.3	0.394	0.4	0.1–1.2	0.113
**Circulatory system**						
Ischaemic heart disease	1.1	1.0–1.4	0.107	0.4	0.3–0.7	<0.001
Heart failure	1.0	0.8–1.3	0.778	0.6	0.5–0.8	<0.001
Cerebrovascular disease	1.0	0.8–1.2	0.870	0.3	0.2–0.6	<0.001
Hypertensive disease	1.1	0.8–1.4	0.667	0.6	0.3–1.1	0.122
**Respiratory system**						
Influenza, pneumonia	0.8	0.7–1.0	0.028	0.5	0.4–0.6	<0.001
Chronic lower respiratory disease	1.1	0.8–1.3	0.591	0.7	0.4–1.2	0.181
Asthma	1.1	0.5–2.5	0.795	0.2	0.0–1.2	0.076
Pneumonitis due to solids/liquids	0.9	0.7–1.2	0.558	0.6	0.5–0.8	<0.001
**Digestive system**						
Intestinal obstruction	0.6	0.3–1.1	0.101	0.3	0.1–0.7	0.005
Cirrhosis /liver disease	1.0	0.8–1.2	0.719	0.5	0.3–0.6	<0.001
**Genitourinary system**						
Renal diseases/dialysis	1.1	0.8–1.5	0.522	0.6	0.3–1.2	0.153
**Signs and symptoms**						
Ill-defined/ unknown	1.3	0.4–4.4	0.710	0.6	0.4–1.0	0.038
**Injury and poisoning**						
Intentional self-harm	1.5	1.0–2.1	0.035	1.3	0.8–1.9	0.285
Accidental falls	0.8	0.5–1.5	0.560	0.7	0.4–1.1	0.135
Accidental poisoning^1^	0.9	0.7–1.3	0.599	0.8	0.5–1.3	0.372
Choking on food/solids^2^	3.7	1.6–8.6	0.003		NE	

†Hazard ratios adjusted for age, sex, accessibility, relative disadvantage, indigenous status, partner status, country of birth and number of comorbid conditions.

^**¥**^See Table 2 for the number of decedents included in each cause of death subset regression model. The clustered sandwich estimator was used to account for clustering of events and strata weights applied. Proportional hazards assumed.

^1^Includes poisoning by analgesics, antipyretics, antirheumatics, narcotics, psychodysleptics, antiepileptic, sedative-hypnotic, anti-parkinsonism or psychotropic drugs.

^2^ Inhalation and ingestion of food causing obstruction of respiratory tract. NE, not estimable due to small numbers with this cause of death.

### Hospital admissions

In general, decedents with a history of schizophrenia demonstrated less frequent hospital admissions compared to matched comparison decedents, but they tended towards staying in hospital longer once admitted (Table 4). Relatively fewer females but not males with schizophrenia were admitted to hospital at least once compared to their gender matched cohort. Hospital admissions by decedents with schizophrenia were more often for psychogeriatric care, maintenance care and rehabilitation but less often for acute care or palliative care. Of decedents with schizophrenia who were admitted to hospital, more were emergency admissions, involved more days of psychiatric care, involved more intensive care (males only) or ventilator support (females only).

**Fig 2:**
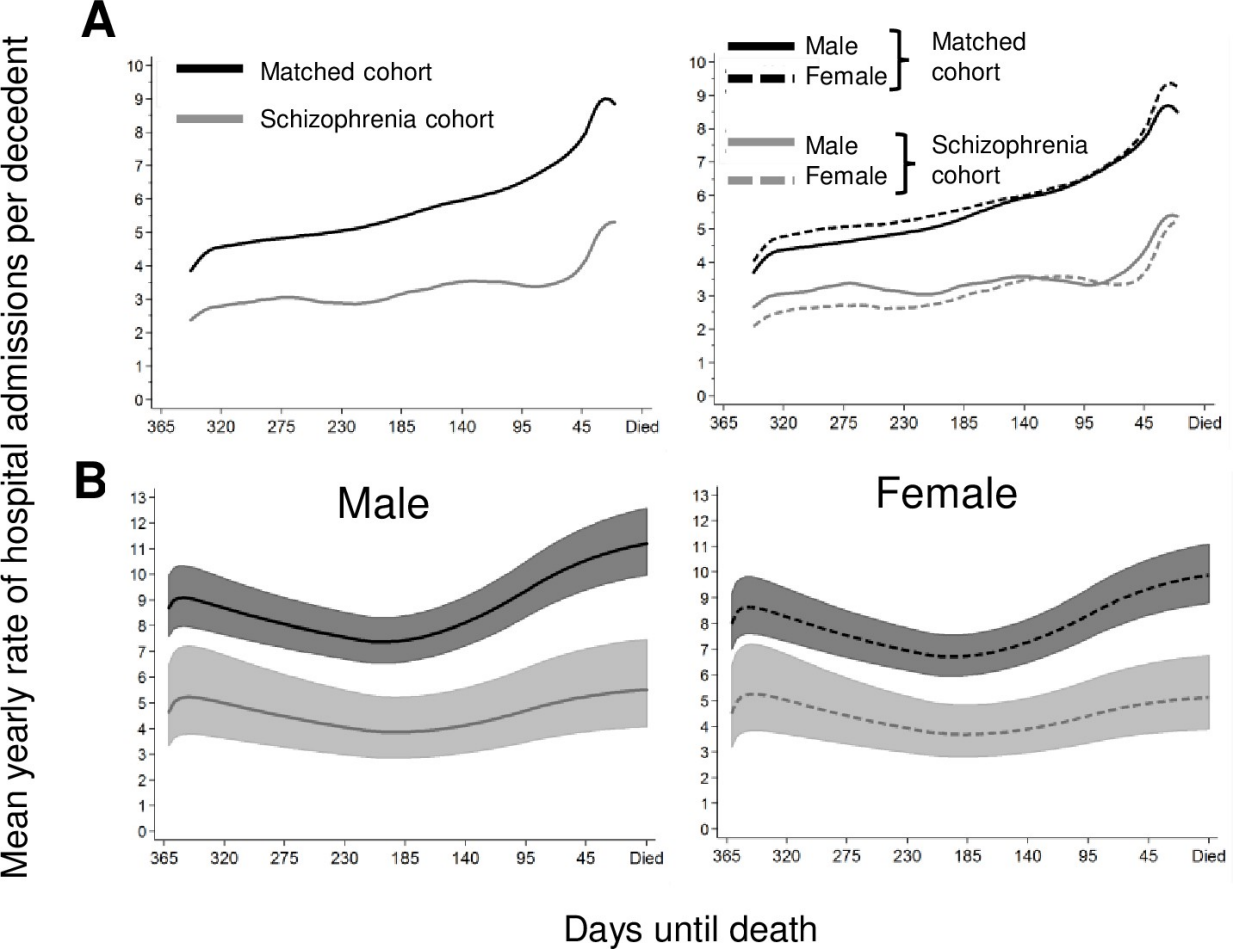
**The average yearly (hazard) rate of hospital admissions per decedent over the last year of life for the schizophrenia and matched cohorts and stratified by sex estimated from A) an unadjusted kernel-weighted estimator of the hazard and B) a flexible parametric proportional hazards model adjusted for age at death, comorbidity, socioeconomic status, accessibility index, sex, partner status, indigenous status and country of birth.** Hazard rates and 95%CI in B are those predicted for Australian born non-Indigenous decedents living in a metropolitan area of average disadvantage without a partner, aged 50 years or older at death and with three comorbid conditions.

The average unadjusted rate of hospital admissions for the schizophrenia cohort was consistently lower than the matched comparison cohort over the last year of life (Fig 2A) a trend that was consistent for both males and females. After adjusting for confounders, the rates of hospital admission over the last year of life for decedents with schizophrenia remained consistently lower for both females and males than their gender matched cohort (Average HR 0.53; 95%CI 0.44–0.65).

Rates of hospital admission generally showed a lower trend in decedents with schizophrenia for almost all causes of death, although limited statistical power was present in less common causes of death. Overall, decedents with schizophrenia who died from cancer had 40% lower rates of hospital admission in the last year of life compared to the matched cohort who also died from cancer, except for breast cancer where admission rates were similar. Lower rates of hospital admissions in the last year of life were observed for decedents with schizophrenia who had infections (influenza, pneumonia or bacterial sepsis), diseases of the circulatory system (ischaemic heart disease, heart failure and cerebrovascular disease), liver disease and pneumonitis recorded on their death certificate compared to decedents in the matched cohort with the same conditions. There were no causes of death where rates of hospital admission by decedents with schizophrenia was higher than the matched cohort, except a non-significant increase for those who died from intentional self-harm.

### Community-based care in the last year of life

Just under 7% of all decedents with schizophrenia accessed community-based specialist palliative care in the last year of life compared to 16% of decedents in the matched cohort (Table 6). When restricted to decedents who died from conditions considered amenable to palliative care around 12% of decedents with schizophrenia and 25% of the matched cohort received community-based specialist palliative care. Decedents with cancer accessed the greatest proportion of community-based specialist palliative care in both cohorts.

**Table 6:** Summary of community-based non-palliative and specialist palliative care accessed in the last year of life by the schizophrenia and matched cohorts.

Community-based care accessed in the last year of life	Schizophrenia Cohort N = 1194		Matched Cohort† N = 39265		
	N	%	N	%	P-value
**Specialist palliative care**					
Registered decedents	78	6.5	6217	15.8	<0.001
Total hours care per decedent (mean, se)	13.2	2.3	23.3	0.8	<0.001
No. (%) decedents with COD amenable					
to palliative care who received SPC‡	74	11.9	5831	24.7	<0.001
By specific causes of death					
Cancers	60	27.5	5324	40.4	<0.001
Heart failure	8	5.8	286	7.5	0.472
Renal disease	5	5.6	415	11.1	0.122
Chronic lower respiratory disease	10	7.3	397	12.8	0.066
Dementias	<5	2.6	209	5.9	0.111
Cirrhosis and liver failure	<5	9.1	264	13.0	0.494
Parkinson’s disease	<5	3.9	22	5.3	0.781
**Non-palliative care**					
Registered decedents	528	44.2	16331	41.6	0.075
Services accessed					
Allied health care (centre)	7	0.6	236	0.6	0.951
Allied health care (home)	77	6.5	2564	6.5	0.913
Assessment	468	39.2	14567	37.1	0.148
Centre based day care	132	11.1	3243	8.3	<0.001
Client care coordination	333	27.9	9456	24.1	0.003
Counselling (client)	162	13.6	4363	11.1	0.009
Counselling (carer)	111	9.3	2576	6.7	<0.001
Domestic assistance	245	20.5	8148	20.8	0.847
Home maintenance	115	9.6	3678	9.4	0.761
Nursing care (home)	175	14.7	5499	14.0	0.530
Nursing care (centre)	12	1.0	360	0.9	0.761
Personal care	159	13.3	4440	11.3	0.034
Respite care	29	2.4	1115	2.8	0.403
Social support	200	16.8	4295	10.9	<0.001

† Coarsened exact matching strata weights applied and p-values corrected to account for study design.

‡ Causes of death amenable to palliative care were defined as occurring anywhere on the death certificate. Decedents could have had more than one causes of death amenable to palliative care which is why the total exceeds the number of decedents registered for the service. Numbers less than five are suppressed. SPC, specialist palliative care; COD, cause of death; se, standard error.

The relative rate of ED presentation and hospital admissions during periods of time of receiving community-based specialist palliative care compared to periods of time not receiving this care were investigated on the subset of decedents with conditions amenable to palliative care (Table 7). Amongst decedents with schizophrenia, days enrolled in community-based specialist palliative care was associated with no change in rate of ED presentations and a 50% increase in rate of hospital admissions. Amongst decedents in the matched cohort, periods of time enrolled in the service was associated with reduced rates of ED admissions but no difference in rate of hospital admissions.

**Table 7:** Association of community-based specialist palliative care with rates of ED presentations and hospital admissions for the schizophrenia and matched cohorts amongst the subset of decedents who died from conditions amenable to palliative care (n = 18,946).

Decedents with cause of death amenable to palliative care^¥^	ED presentations†			Hospital admission†		
	HR	95%CI	p	HR	95%CI	p
Receiving SPC versus no SPC care within						
Schizophrenia cohort (n = 425)	1.2	0.8–1.7	0.372	1.5	1.1–2.1	0.011
Matched cohort (n = 18521)	0.8	0.7–0.9	<0.001	1.0	0.9–1.1	0.649
Schizophrenia vs matched cohort within						
Days enrolled in SPC care	1.6	1.2–2.4	0.007	0.8	0.6–1.0	0.041
Days not enrolled in SPC care	1.1	1.0–1.4	0.101	0.5	0.4–0.7	<0.001

†Relative hazard (rates) adjusted for age, sex, accessibility index, relative disadvantage, indigenous status, partner status, country of birth and number of Elixhauser comorbid conditions.

^**¥**^Cancers, organ failures, dementia, Parkinson's disease and chronic obstructive pulmonary disease. The clustered sandwich estimator was used to account for intra-decedent clustering of events and strata weights applied. SPC, community-based specialist palliative care; COD, cause of death; ED, emergency department; HR, hazard rate.

Around 42% of decedents in both the schizophrenia cohort and matched cohort were registered as clients of community-based (non-palliative) care services in last year of life. Decedents with schizophrenia were more likely to have received centre-based day care, counselling for both client and carer, social support, personal care and client management services compared to the matched cohort.

## Discussion

We report that health service use in the last of life for people with schizophrenia living in Western Australia is very different to that experienced by people without schizophrenia. People with schizophrenia died from different causes of death, had unique patterns of ED use, were half as likely to enrol in community-based specialist palliative care and were half as likely to be admitted to hospital in the last year of life compared to people without schizophrenia who had a similar sociodemographic profile.

### Causes of death

The leading causes of death for people with schizophrenia were cancers, diseases of the circulatory system, intentional self-harm and accidental poisoning (drug overdoses) compared to an age-matched cohort, patterns similar to that reported in Canada [36], United States [4] and Sweden [37]. Deaths from epilepsy were overrepresented in decedents with schizophrenia. Temporal lobe epilepsy has long been associated with a schizophrenia-like psychosis although understanding of the neuropathological and temporal mechanisms remain unclear. [38] Just over 2% of all decedents with schizophrenia had Parkinson’s disease recorded as the underlying or a contributory cause of death. Coexistence of schizophrenia with Parkinson’s disease is generally considered a rare condition with the hyperactive dopamine transmission observed in schizophrenia considered oppositional to the dopaminergic deficiency underlying Parkinson’s disease. [39] It is possible that drug-induced Parkinsonism, the result of anti-psychotic medication, was misclassified as Parkinson’s disease in most cases in our study.

The over representation of death from pneumonitis due to solids/liquids and choking in decedents with schizophrenia are not unexpected as disorders of swallowing are common and result from both the illness and the medication used to treat psychotic disorders [40]. An increased number of deaths with intestinal obstruction were observed in decedents with schizophrenia, possibly associated with gastrointestinal hypomotility induced by some antipsychotic agents, such as clozapine. [41]

### ED use in last year of life

The association of schizophrenia with rate of ED use in the last year of life was complex and varied with sex and comorbidity. Within the subset of decedents without a history of comorbidity, males with schizophrenia were the highest users of ED. The greater proportion of ED presentations involving police or corrective services and symptoms related to alcohol and drug abuse and behavioural problems in males with schizophrenia reflects common psychiatric comorbidities in this group. Decedents with schizophrenia who died of intentional self-harm presented to ED 50% more often in the last year of life than decedents without schizophrenia who also died from self-harm. More ED presentations for social/behavioural and drug and alcohol problems in people with schizophrenia who died from suicide suggests less stable schizophrenia and consequences of psychosis. However, as decedents with schizophrenia who died with cancer also had higher rates of ED presentation compared to the matched cohort, reasons for increased ED use are likely multifactorial and include patient, provider and system processes. People with schizophrenia and cancer may find it difficult to communicate medical history and health care providers may be more likely to attribute medical symptoms to the schizophrenia [9], potentially leading to repeat ED presentations.

In the subset of decedents who were dying with conditions amenable to palliative care, we observed that periods of time receiving community-based specialist palliative care was associated with reduced ED presentations for the matched cohort in the last year of life, as reported previously in Western Australia. [15] However, this was not the case in decedents with schizophrenia where no change in the rates of ED presentation during periods receiving and not receiving community-based specialist palliative care were observed. We speculate that contact with a specialist palliative care change was likely to have influenced the motivators of ED presentation in people with schizophrenia, however our data lacked the clinical detail and statistical power to identify if any subtle changes in the reasons for ED presentations during times with and without palliative care had occurred.

### Hospital use in the last year of life

Overall the rate of hospital admission in the last year of life was 50% reduced in decedents with schizophrenia, larger than the 27% reduction in rate of hospital separations by people with schizophrenia in the last six months of life reported in a Canadian study. [12] This difference could partly be explained by methodology as we excluded person-time at risk while hospitalised.

Reduced rates of hospital admission for people with schizophrenia were evident across most causes of death with the strongest evidence observed for infections, cancers, most circulatory system diseases, dementias, depression, alcohol abuse and liver disease. The only causes of death where rates of hospital admission were similar between the cohorts were for breast cancer and intentional self-harm. While the average length of hospital stay was longer and involved more days of psychiatric care for decedents with schizophrenia, reduced rates of hospitalisation likely represent the same complex interaction of patient, individual clinician and healthcare system factors that contribute to disparate outcomes in other marginalised groups.

In the subset of decedents with schizophrenia who also had conditions amenable to palliative care, being enrolled in community-based specialist palliative care was associated with a 50% increase in rates of hospital admission. This finding suggests that receiving home-based specialist palliative care is reducing barriers to accessing hospital care. However, our data were insufficiently detailed to determine whether this increase reflected appropriate hospitalizations or instead reflected reduced access to appropriate care elsewhere in the community. Further work is required to address this issue.

### Access to community-based care

We found evidence that people with schizophrenia who are dying with conditions amenable to palliative care were half as likely to receive specialist palliative care in the home compared to the matched cohort. This is similar to findings from New Zealand [42] and Canada. [12] Yet, we found that decedents with schizophrenia were just as likely to have accessed non-palliative community-based services in the last year of life as decedents without schizophrenia. Studies from countries with universal health care like Australia have also reported regular and increased access to GP services by people with schizophrenia. [12, 43] This suggests the barriers inhibiting receipt of palliative care for people with schizophrenia are at least partly health system driven rather than patient driven. Considering the vulnerability of people with schizophrenia to poorer general health outcomes and lower life expectancy, efforts to remedy this are essential.

### Study strengths and limitations

Strengths of this study were the population based source of participants and record linkage across multiple different datasets. Limitations of the study included lack of clinical information and certainty related to schizophrenia diagnosis, particularly when coded in emergency department data or as a comorbid condition in hospital records. Our cohort of decedents with schizophrenia were selected from administrative records dating back to 1970 so misclassification of older decedents with schizophrenia who only had hospital records prior to this time was possible. It is also possible that comorbidity was either reported less frequently on medical records or was underdiagnosed for people with schizophrenia possibly leading to some differential bias. Lack of accurate date of service data for the community-based non-palliative care limited interpretation. Our methodological approach involved multiple regression models, thus interpretation of findings raises the issue of multiple comparisons. We did not make statistical adjustment to account for multiple testing in this study based on the view that readers will take the design of the study, the associated effect sizes, support from other published studies and our conservative interpretation into account. We also note that for less frequent failure events and small cause of death categories, the statistical power was only sufficient to detect very large differences between the two cohorts and that false positive findings were also possible.

In the last year of life, people with schizophrenia were less likely to be admitted to hospital, more likely to attend ED if male, less likely to access community-based speciality palliative care and had a different comorbidity and cause of death burden compared to matched decedents with a similar sociodemographic profile. Community-based specialist palliative care was associated with increased rates of hospital admission in decedents with schizophrenia. Our findings suggest that accessing community-based specialised palliative care can provide an alternative path to hospital care for persons with schizophrenia dying from conditions amenable to palliative care during the last year of life.

## Supporting information

S1 Table ICD-10-AM codes used to identify cause of death groups. (DOCX)
