# Supplementary material for: Morbidity burden and community-based palliative care are associated with rates of hospital use by people with schizophrenia in the last year of life: A population-based matched cohort study
Source: PLoS One. 2018 Nov 29;13(11):e0208220. doi: 10.1371/journal.pone.0208220 (PMC6264825; doi:10.1371/journal.pone.0208220)
Supplement: S1 Table — (DOCX) [file pone.0208220.s001.docx]

S1 Table: ICD-10-AM codes used to identify cause of death groups.

| **Cases of death** | **ICD-10 codes** |
| --- | --- |
| **Certain infectious diseases** |  |
| Bacterial sepsis | A40, A41 |
| **Neoplasms** |  |
| Lung cancer | C33, C34 |
| Breast cancer | C50 |
| Colorectal cancer | C18-C21 |
| Blood & lymph cancers | C81-C95 |
| Malignancy of ill-defined sites | C76-C80 |
| All other cancers /chemotherapy | Remaining C codes, D00-D48, Z511 |
| All cancers | All C codes, , D00-D48, Z511 |
| **Endocrine system** |  |
| Diabetes | E10,E11, E13, E14 |
| **Mental/behavioural disorders** |  |
| Dementias | F01, F03, G30 |
| Due to use of alcohol | F10 |
| Due to use of drugs | F11-F16, F18,F19 |
| Schizophrenia | F20, F21, F231, F232, F25 |
| Bipolar affective disorders | F31 |
| Depressive episodes | F32, F33 |
| **Nervous system** |  |
| Epilepsy | G40, G41 |
| Parkinson's disease | G20 |
| **Circulatory system** |  |
| Hypertensive disease | I10-I15 |
| Ischaemic heart disease | I20-I25 |
| Heart failure | I50, I51 |
| Cerebrovascular disease | I60-I69 |
| **Respiratory system** |  |
| Influenza, pneumonia | J09 -J18 |
| Chronic lower respiratory disease | J40-J44 J47 |
| Asthma | J45 J46 |
| Pneumonitis due to solids, liquids | J69 |
| **Digestive system** |  |
| Intestinal obstruction | K56 |
| Cirrhosis and liver disease | K70-K76 |
| **Genitourinary system** |  |
| Renal diseases/renal dialysis | N00-N19, N25-N29, Z49 |
| **Signs and symptoms** |  |
| Ill-defined/ unknown cause of mortality | R99 |
| **Injury and poisoning** |  |
| Intentional self-harm | X60-X84 |
| Accidental falls | W00-19 |
| Accidental poisoning (drugs/noxious substances) | T36-T65, X40-X49 |
| Choking on food/liquid | W79 |
